# GNSS performance enhancement using measurement estimation in harsh environment

**DOI:** 10.1371/journal.pone.0292116

**Published:** 2023-09-27

**Authors:** Jae Hwan Bong, Doyoung Kim, Seongkyun Jeong

**Affiliations:** Department of Human Intelligence Robot Engineering, Sangmyung University, Cheonan, Korea; ICIMOD: International Centre for Integrated Mountain Development, NEPAL

## Abstract

Global navigation satellite systems (GNSSs) are commonly used to measure the position and time globally. A GNSS is convenient owing to its ability to measure accurate position relatively without using assistive tools for navigation by comparing with other sensors. Based on these benefits, the applicable area is expanding to commercial and social uses (e.g., vehicle navigation, smart grids, and smartphone apps). In the future, various services and technologies (e.g., the use of autonomous vehicles, unmanned delivery, and industrial field robots), which make Internet of Things (IOT) more active, will be used in our society. Conversely, the performance of GNSS can degrade in harsh environments, such as urban areas, owing to the property of GNSS, which calculates position and time via satellite signal reception. However, buildings in a city can block navigation satellite signals and generate multi-path errors. The blocked signals exacerbate the dilution of precision (DOP), which indicates the accuracy of the navigation solution and increases the navigation solution error. This study proposes methods to improve navigation performance by leveraging various techniques (e.g., range differences, receiver clock error hold, and virtual satellites). The methods were validated in harsh environments where visible satellites were reduced. In the simulation, each proposed method improved the navigation performance by creating an environment similar to a normal situation, despite the receiver entering a harsh environment. The results confirmed that the navigation performance deteriorated compared to the normal situation where the number of visible satellites decreased. However, the navigation performance was recovered gradually by applying the proposed techniques. Using the proposed methods, navigation performance can be maintained continuously even in situations where satellite signals are blocked.

## Introduction

The global navigation satellite system (GNSS) provides the receiver’s position and time information owing to navigation satellite signals. Conversely, military, commercial, and social uses of GNSSs have increased substantially. In the future, various uses of GNSSs will be accelerated in areas that apply Internet of Things (IOT), such as autonomous driving and unmanned delivery. Unmanned mobile technology will not only increase commercial value and convenience, but also help disabled people, thereby increasing the use of GNSS in urban areas. Furthermore, a reliable navigation system requires a long mission range and high accuracy [1].

Despite the convenience of obtaining navigation information without using initial positioning, other types of sensor information, or the continuous accumulation of position errors, GNSS faces severe problems in harsh environments such as cities and canyons. The signal strength of GNSS is very weak, and easily affected and blocked by the surrounding environment where the navigation signals are blocked. Therefore, the navigation performance of GNSS is degraded [2]. When satellite signals are blocked by high obstacles, the navigation receiver must calculate the navigation solution using only high-elevation satellites. When the receiver receives only the satellite signal around the zenith, the position is calculated using satellite measurements from limited areas, even if it has the same number of satellites, resulting in lower accuracy and increased dilution of precision (DOP) values [3]. Furthermore, the continuity of the navigation solution, compared with the previously calculated values, breaks immediately, making it difficult to utilize GNSS and trust and use GNSS in systems that require high reliability and, high-level position information stability, such as autonomous vehicles and unmanned delivery drones [4].

Studies have been conducted for overcoming harsh environments in urban areas. A study applied 3D modeling. Because the signal is blocked along the street in the downtown area, this method estimates the location by applying 3D modeling [5, 6]. This method improved the navigation performance for receivers in urban areas including smart phones [7]. A machine learning technique was introduced to distinguish between blocked and visible satellites [8], and a technique was proposed to maintain satellite navigation performance by improving the robustness of signal processing [9]. These assistant navigation sensors or map information help overcome the increasing navigation errors in harsh environments, however, it complicates the structure of the navigation system [10]. Therefore, technology that maintains the navigation performance even when satellite navigation receivers enter harsh environments helps increase the utilization, reliability and convenience of GNSSs. This study proposed techniques to generate effects that enhance visibility and maintain navigation performance even in harsh GNSS environments.

Several studies have been conducted to maintain navigation performance in harsh environments where visible satellites are reduced while using GNSSs [11–13]. One of these methods is to correct the clock error of the receiver which increase the number of visible satellites by reducing the unknown parameter, that is the receiver clock error. This parameter is determined when the receiver calculates the navigation solution. The receiver clock error does not change significantly in the continuous visible-satellite combination. If the satellite signals are partially blocked, the position and time are calculated using the remaining visible satellites. The probability of the receiver clock error changing increases. Based on the situation that the receiver clock error does not change significantly, this method maintains the receiver clock error as the number of visible satellites decreases, thereby obtaining additional visible satellite effect [14].

Another method is to increase the number of satellites by placing a virtual satellite far away from an actual satellite [15]. The navigation solution is calculated using the pseudorange of a virtual satellite. The deployment of virtual satellites further than conventional satellites results relatively little pseudorange change compared to the motion of the receiver, and improves the geometric arrangement of the satellite. Consequently, the DOP value is enhanced. Therefore, it can be used to improve accuracy [16].

Recently, various navigation systems such as Beidou, Galileo, and GLONASS, have been developed. Methods of calculating the navigation solution using multi-GNSS systems have been studied [17, 18] and the error factors decrease as GNSS signals become more precise [19]. Because of the increasingly precise GNSS signal, it is actively used in various forms, such as earth geodesy and earthquake research [20].

Although these technologies help improve navigation performance, problems still persist. For a technique to hold the receiver clock error, it is difficult to maintain continuity between the navigation solutions calculated from previous measurements and from reduced visible satellites.

Even if the clock error of a receiver is held, the receiver’s position is calculated using the relationship between visible satellite measurements making it difficult to maintain consistency with previous navigation solution. The navigation solution using pseudoranges that reflects the error factors of the current visible satellites is different from the navigation solution using previous pseudoranges. A method that improves the DOP performance by applying virtual satellites cannot maintain navigation continuity considering virtual satellites are considered new satellites. In particular, applying this method in the presence of reduced visible satellites makes it difficult to compute a virtual satellite’s pseudorange in situations where the satellite is created in a new location, because the exact position of the receiver is unknown. The application of random pseudoranges generates many errors. From the receiver’s perspective, virtual satellites are recognized as additional new satellites, indicating the effectiveness of calculating the receiver position with changed measurement inputs. Consequently, existing methods have limitations in terms of improving the positional errors for reducing visible satellites. Although multi-GNSS can acquire many satellites, the satellite locations are limited in urban areas.

When satellite navigation receivers enter harsh environments and the number of received satellites decreases, the position accuracy of the receiver is degraded. When the signals from the satellite and measurements are reduced, the receiver’s navigation solution changes, and the error increases. A fundamental solution for reducing this generated error is to return to the environment before the receiver enters the harsh environment. In this study, a method in which the receiver calculates a navigation solution by reflecting the environment as much as possible before the error occurs is proposed to reduce the error in the navigation solution. To realize this concept, the range difference, receiver clock error hold, and virtual satellite methods were proposed. However, the enhancement of the navigation performance depends on the number and arrangement of navigation satellites owing to the properties of the navigation solution calculation method. Therefore, because the overall performance should be verified based on various receiving environments, the performance at the algorithm level was verified by validating the performance improvement while applying each method stepwise.

The degradation of navigation performance in harsh urban environments is due to the decrease in the number of visible satellites. In this environment, GNSS availability dwindles, resulting in decreased position accuracy and disrupted continuity. In the mentioned methods, the performance degradation is solved as an additional equipment, but the cost and complexity of the system increase. This study takes a different approach, focusing on addressing the root causes of the problem through software-based solutions. Our methodology aims to preserve accuracy and continuity by leveraging existing navigation solutions. Given that the disappearance of visible satellites in urban areas varies depending on the configuration and placement of obstacles, it is impractical to validate performance across all environments and scenarios. This study takes a different approach, focusing on addressing the root causes of the problem through software-based solutions. Our methodology aims to preserve accuracy and continuity by leveraging existing navigation solutions. Given that the disappearance of visible satellites in urban areas varies depending on the configuration and placement of obstacles, it is impractical to validate performance across all environments and scenarios. Hence, in this study, we conducted simulations under specific conditions to verify the effectiveness of the proposed algorithm. While the algorithm’s verification was conducted in a limited environment, it is crucial to note that environments characterized by the disappearance of visible satellites are precisely where navigation solution performance degradation occurs. The efficacy of our proposed algorithms can be demonstrated by comparing their performance to that of previous methods.

## Materials and methods

Various methods have been developed to increase navigation performance, including differential-GNSS(D-GNSS) and real-time kinematics (RTK) techniques. The D-GNSS technique improves the navigation performance by differentiating the error components of the measured values, while the RTK technique improves the performance by using a reference station and rover receiver. The RTK method is appropriate for precise positioning and is generated by collecting RTK correction signals from multiple reference stations located near the user’s location. Then, they are used comprehensively to calculate the RTK correction signal suitable for the user’s location. RTK also improves its performance in various ways, including traditional RTK, long base RTK (LBRTK), network RTK (NRTK), and precise point positioning RTK (PPP-RTK). NRTK and LBRTK are popular owing to their distance, time, and accuracy [21–23]. Therefore, the reliability must be guaranteed to use these techniques.

When a navigation receiver receiving GNSS signals in a normal environment moves to a harsh environment the interrelationship between measurements changes in navigation solution calculations. Consistency between measurements is crucial for maintaining navigation performance in harsh environments and to reduce differences from previous navigation solutions. The position and time output is the result of aggregating the measurements of visible satellites considering the measurements on the navigation receiver contain errors, and multiple measurements are used to obtain the final solution. When a receiver is placed in a harsh environment, the number of visible satellites decreases and the position and time change depending on the interrelationship with the reduced visible satellites. In particular, applying satellites with high-elevations reduces the DOP value and increases navigation errors.

This study focused on the navigation solution characteristics that can improve performance by correcting the error component in the pseudorange. The proposed methods maintain consistency in the relationship between satellite measurements and improve the degraded navigation performance.

The first method calculates the range difference for each measurement and applies it to the navigation solution to maintain consistency. When the number of visible satellites decreases in harsh environments, only visible satellites are used to calculate navigation solutions, and the receiver has an error compared with previous navigation values. To reduce this error, pseudorange measurement errors on existing visible satellites are monitored and the monitored values are used even when entering harsh environments. The estimated range calculated using the range between the calculated navigation solution and the position of the satellite. Eq (1) calculates the estimated range.

$$r_{ER}=\sqrt{{\left(x_{i}-x_{r}\right)}^{2}+{\left(y_{i}-y_{r}\right)}^{2}+{\left(z_{i}-z_{r}\right)}^{2}}$$ (1)

where *r*_*ER*_ is the estimated range, (*x*_*i*_, *y*_*i*_, *z*_*i*_) is the position of the i-th satellite, and (*x*_*r*_, *y*_*r*_, *z*_*r*_) is the position of the receiver which is calculated with receiver’s measurement.

The range difference is calculated as the difference between the receiver measurement and the estimated range, given as

$$E_{PR}=r_{PR}-r_{ER}$$ (2)

where *E*_*PR*_ is the range difference and *r*_*PR*_ is the measured pseudorange.

The range difference which is the value considering the navigation solution and pseudorange error does not change significantly if a special error is not applied and visible satellites are maintained. However, as the number of visible satellites decreases owing to the transition from normal to harsh conditions, the range difference changes as the number of measurements in the navigation solution calculation decreases. This study proposes a method to compute a navigation solution by reflecting the range difference in the pseudorange measurement. The method of maintaining the range difference to reduce deviation and maintain the continuity of the navigation solution is considered in harsh environments that reduce visible satellites. Eq (3) shows the results that reflect the range differences in the pseudorange.

$$r^{\prime }{}_{PR}=r_{PR}-E_{PR}$$ (3)

where *r*′_*PR*_ is the corrected pseudorange. When calculating the navigation solution using the corrected pseudorange, the navigation solution performance can be maintained using only visible satellites in harsh environments.

δρ=αδx (4)

$$\begin{array}{l} \delta \rho ={\left[\begin{array}{cccc} \delta \rho_{1} & \delta \rho_{2} & \cdots & \delta \rho_{n} \end{array}\right]}^{T}\\ \delta x={\left[\begin{array}{cccc} \delta x_{r} & \delta y_{r} & \delta z_{r} & \delta b_{r} \end{array}\right]}^{T}\\ \alpha =\left[\begin{array}{cccc} \alpha_{11} & \alpha_{12} & \alpha_{13} & 1\\ \alpha_{21} & \alpha_{22} & \alpha_{23} & 1\\ \vdots & \vdots & \vdots & \vdots \\ \alpha_{n1} & \alpha_{n2} & \alpha_{n3} & 1 \end{array}\right] \end{array}$$ (5)

$$\alpha_{i1}=\frac{x_{i}-x_{r}}{\rho_{i}-b_{r}},\alpha_{i2}=\frac{y_{i}-y_{r}}{\rho_{i}-b_{r}},\alpha_{i3}=\frac{z_{i}-z_{r}}{\rho_{i}-b_{r}}$$ (6)

where *ρ* is the pseudorange and *b*_*r*_ is the receiver clock bias.

A range difference occurs between the pseudorange obtained from the actual satellite and the distance from the calculated position to the satellite. This range difference does not change significantly in the case of similar environments such as satellite constellations, visible satellites, and ionosphere errors. When the range difference is applied to the pseudorange, which in turn is applied to the current visible satellite when the number of satellites decreases, the position of the receiver can be calculated similarly to the situation before the decrease in visible satellites. Eq (4) represents a position calculation formula with an improved positioning performance.

The second method involves holding a receiver clock error. This method constantly fixes receiver clock errors when calculating navigation solutions. The receiver clock error does not change abruptly after the receiver starts to calculate the navigation solution. However, because this value is obtained in the navigation solution calculation, at least four pseudorange measurements are required. Holding the receiver clock error adds a visible satellite. Eq (7) represents the pseudorange that reflects the receiver clock error, and Eq (8) represents the error between GNSS time and receiver time.

$$r_{PR}=\sqrt{{\left(x_{i}-x_{r}\right)}^{2}+{\left(y_{i}-y_{r}\right)}^{2}+{\left(z_{i}-z_{r}\right)}^{2}}+(t_{i}-t_{r})$$ (7)

$$dt=t_{i}-t_{r}$$ (8)

where *t*_*i*_ is the GNSS time, *t*_*r*_ is the receiver time, and *dt* is the error in the receiver clock. The receiver clock error is expressed as the product of the speed of light in meters.

If the receiver clock error is fixed with a previous visible satellite, the calculation equation for the navigation solution can be expressed as shown in Eq (9).

R−dt=AX+E (9)

$$R=[\begin{array}{c} r_{1}\\ \vdots \\ r_{n} \end{array}],\;X=[\begin{array}{c} x_{r}\\ y_{r}\\ z_{r} \end{array}],\;A=[\begin{array}{ccc} a_{11} & a_{12} & a_{13}\\ a_{21} & a_{22} & a_{23}\\ \vdots & \vdots & \vdots \\ a_{n1} & a_{n2} & a_{n3} \end{array}],\;E=[\begin{array}{c} e_{1}\\ e_{2}\\ \vdots \\ e_{n} \end{array}]$$ (10)

$$a_{i1}=\frac{x_{i}-x_{r}}{r_{i}},a_{i2}=\frac{y_{i}-y_{r}}{r_{i}},a_{i3}=\frac{z_{i}-z_{r}}{r_{i}}$$ (11)

where *R* is a pseudorange matrix, *A* is a system matrix for the navigation solution calculation, *X* is a receiver position matrix, and *E* is a matrix representing pseudorange errors. The receiver’s position matrix does not have receiver clock error, therefore, if the number of visible satellites is greater than three, the receiver position can be calculated using Eq (9), which is a modification of Eq (4).

The receiver clock error hold method is applied in the pre-navigation calculation phase prior to the application of virtual satellites. If the number of visible satellites decreases, the receiver clock error changes, similar to the receiver position. However, applying the range difference and receiver clock error hold methods simultaneously helps maintain the navigation solution continuity and the effect of additional satellites. When calculating the navigation solution, the position and clock error of the receiver are calculated simultaneously, Therefore, if the clock error is kept constant, the unknown number decreases, and the number of satellites increases. Therefore, if the receiver clock error is maintained, the measurement of the navigation satellite is used only for the position calculation, and the error with the existing navigation solution can be reduced.

The third method involves placement of virtual satellites. In previous studies, the arrangement of virtual satellites is as far away from the receiver as possible to reduce the pseudorange variation and improve the DOP performance. In this study, we placed the virtual satellite in a blocked satellite position for navigation continuity maintenance. To maximize the continuity even when virtual satellites are applied. Eq (12) calculates the pseudorange of the virtual satellite.

$$r_{vi}^{\prime }=\sqrt{{\left(x_{i}^{\prime }-x_{r}\right)}^{2}+{\left(y_{i}^{\prime }-y_{r}\right)}^{2}+{\left(z_{i}^{\prime }-z_{r}\right)}^{2}}$$ (12)

where $r_{vi}^{\prime }$ is the pseudorange of the i-th virtual satellite and $\left(x_{i}^{\prime },y_{i}^{\prime },z_{i}^{\prime }\right)$ is the position of the virtual satellite.

The range difference method is also applied to virtual satellites. Eq (13) represents the corrected pseudorange of a virtual satellite with the applied range difference. The error value in Eq (13) uses the last error monitored before the satellite disappears. The error values are generated for each satellite, and each satellite applies its own error value.

$$r_{ci}^{\prime }=r_{vi}^{\prime }+E_{PR}$$ (13)

where $r_{ci}^{\prime }$ is the corrected pseudorange of a virtual satellite.

Virtual satellites help maintain the same environment even if the receiver enters a harsh environment because it provides the effect of calculating the position of the receiver the same as the existing satellite constellation. In previous studies, the virtual satellites method was utilized to improve the vertical rather than horizontal positioning accuracy by arranging virtual satellites and reflecting the actual distance to the receiver in the calculation equation of the navigation solution [24, 25]. In this study, satellites were placed in position before the receiver entered the harsh environment, and the existing environmental conditions were maintained as much as possible to prevent the degradation of the navigation performance.

Previous methods placed the satellite at a relatively long distance, primarily emphasizing subtle pseudorange variations. In contrast, ut the proposed methods focused on maintaining continuity and fixes the virtual satellite at the position where the satellite disappeared. The method was adopted to maintain the environment before the satellite disappeared by maintaining the pseudorange error in the position. When implementing the proposed method, the calculation environment of the navigation solution closely resembles the conditions preceding the satellite’s disappearance. As a result, the performance of the navigation solution can be effectively sustained.

Finally, all the computed pseudoranges are combined to calculate the navigation solution. In the computational phase, a navigation solution is generated by the corrected pseudoranges of visible satellites and the pseudoranges of virtual satellites are generated based on this receiver position. The navigation solution is re-calculated using virtual satellites. This method has a weighting effect on visible satellites and improves the DOP performance in terms of position accuracy. Eq (14) shows an expression for obtaining a navigation solution using the pseudoranges of a virtual satellite.

$$R_{c}=A_{c}X_{c}+[\begin{array}{c} 1\\ 0 \end{array}]E$$ (14)

$$R_{c}=[\begin{array}{c} r_{i}\\ {r^{\prime }}_{ci} \end{array}],\;X_{c}=[\begin{array}{c} x\\ y\\ z\\ \Delta t \end{array}]$$ (15)

where *R*_*c*_ is a corrected pseudorange matrix, *A*_*c*_ is a system matrix for computing navigation solutions using corrected pseudoranges, and *X*_*c*_ is a matrix of the receiver position and clock error.

**Fig 1:**
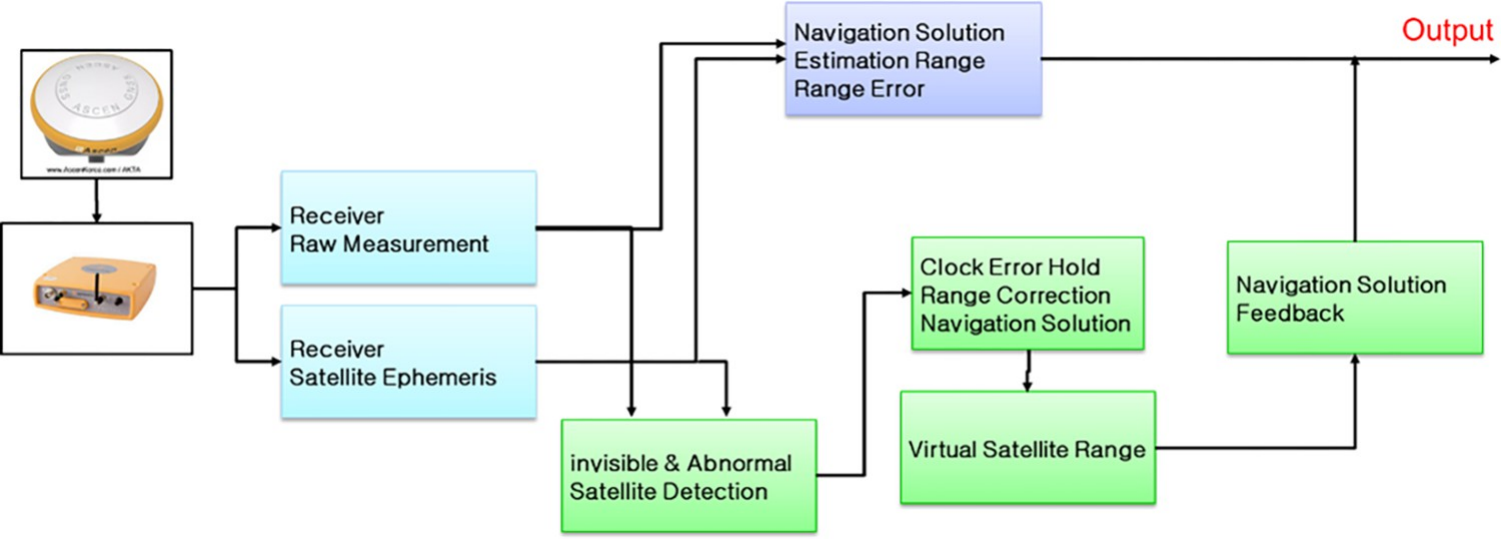
Navigation solution calculation logic using the proposed methods.

Fig 1 shows the logic of computing navigation solutions based on the proposed methods. When the navigation signals are received through the antenna, the receiver extracts the measurement values and satellite information. A navigation solution is calculated and a receiver generates an output based on visible satellite measurements under normal conditions. Furthermore, it calculates the estimated ranges and continues to calculate the range differences to prepare for harsh environment. When satellite signals are blocked in harsh environments, the logic uses pre-calculated range differences and receiver clock errors to calculate the navigation solution. Subsequently, the pseudoranges of the virtual satellites are generated to recalculate the navigation solution in a harsh environment.

In a real environment, the receiver continuously calculates the range difference of the pseudorange. As the range difference is continuously monitored, we confirmed that the value of the range difference was constantly maintained when calculating the navigation solution of the receiver. Because the clock error of the receiver can also be monitored before entering a harsh environment, it can be operated if the satellite signal is blocked when entering a harsh environment. Virtual satellites can be applied at the user’s discretion, and hence can be applied at any time according to the user’s choice. However, if the environment remains harsh, the error of the harsh environment can be maintained, thereby causing an error drift compared with the actual position. In practical application, the proposed method is implemented by reprocessing the receiver’s measurements to minimize positional errors. The receiver’s position is initially computed using the raw measurements it receives, and the associated error is subsequently determined. Continuous error monitoring is an integral part of this process, with the proposed method being activated upon detecting variation in the visibility of satellites. As a result, this approach is suitable for receivers equipped with the capability to access raw measurements, and it can be seamlessly integrated through software modifications, negating the need for additional hardware components. This proposed technique can be effectively applied by employing the algorithm we have introduced to enhance the functionality of existing receivers.

## Result

**Fig 2:**
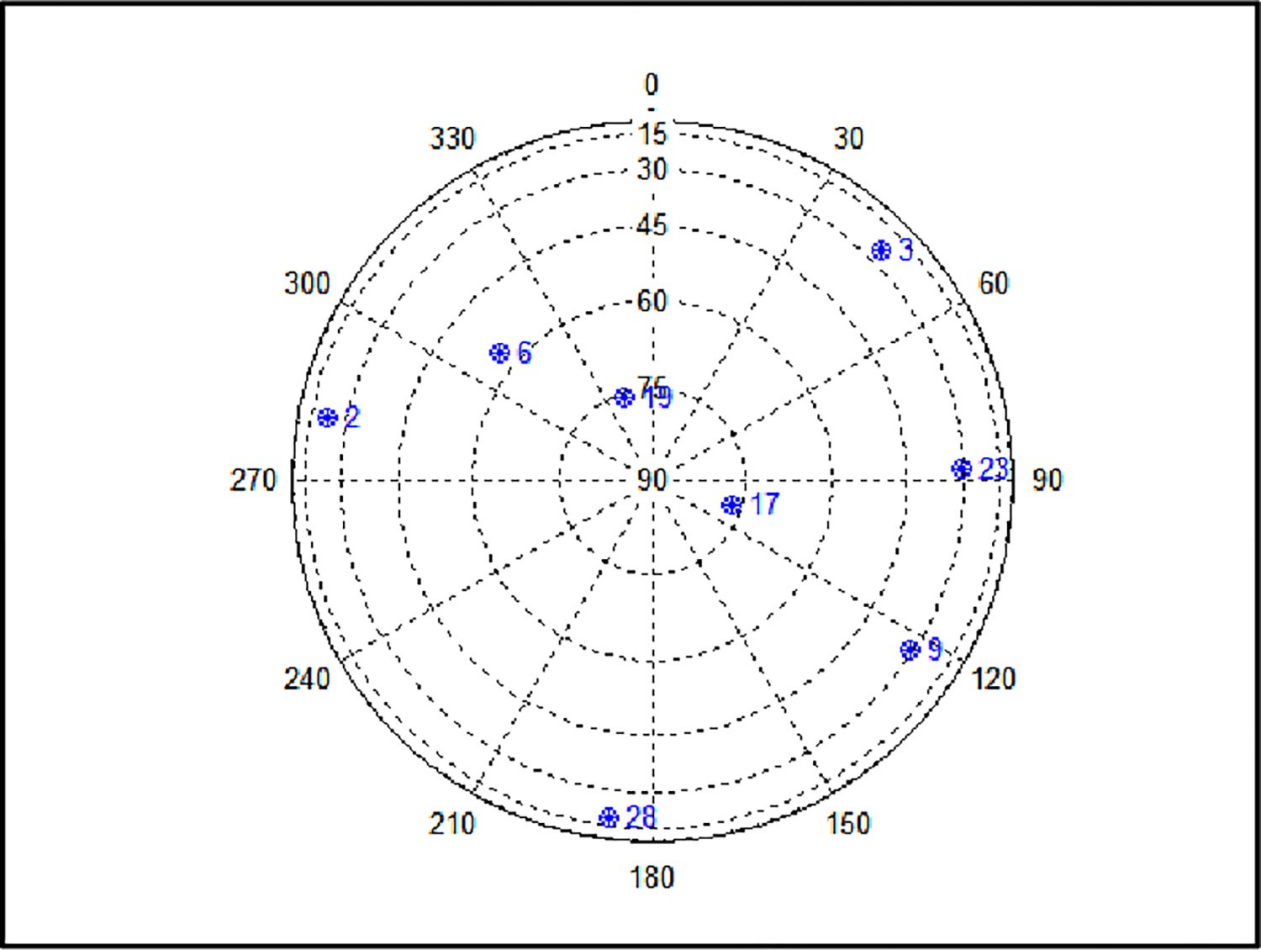
Visible satellite sky plot in normal environment.

**Fig 3:**
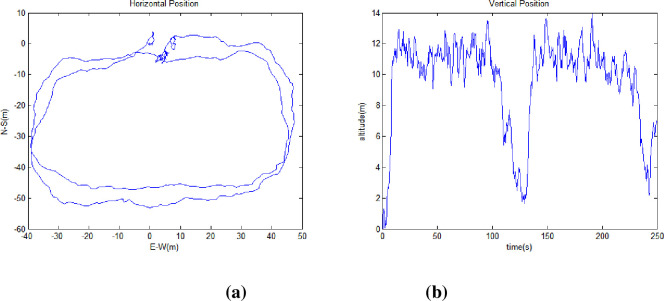
Receiver position in normal environment. (a) Receiver Horizontal Position (b) Receiver Vertical Position.

**Fig 4:**
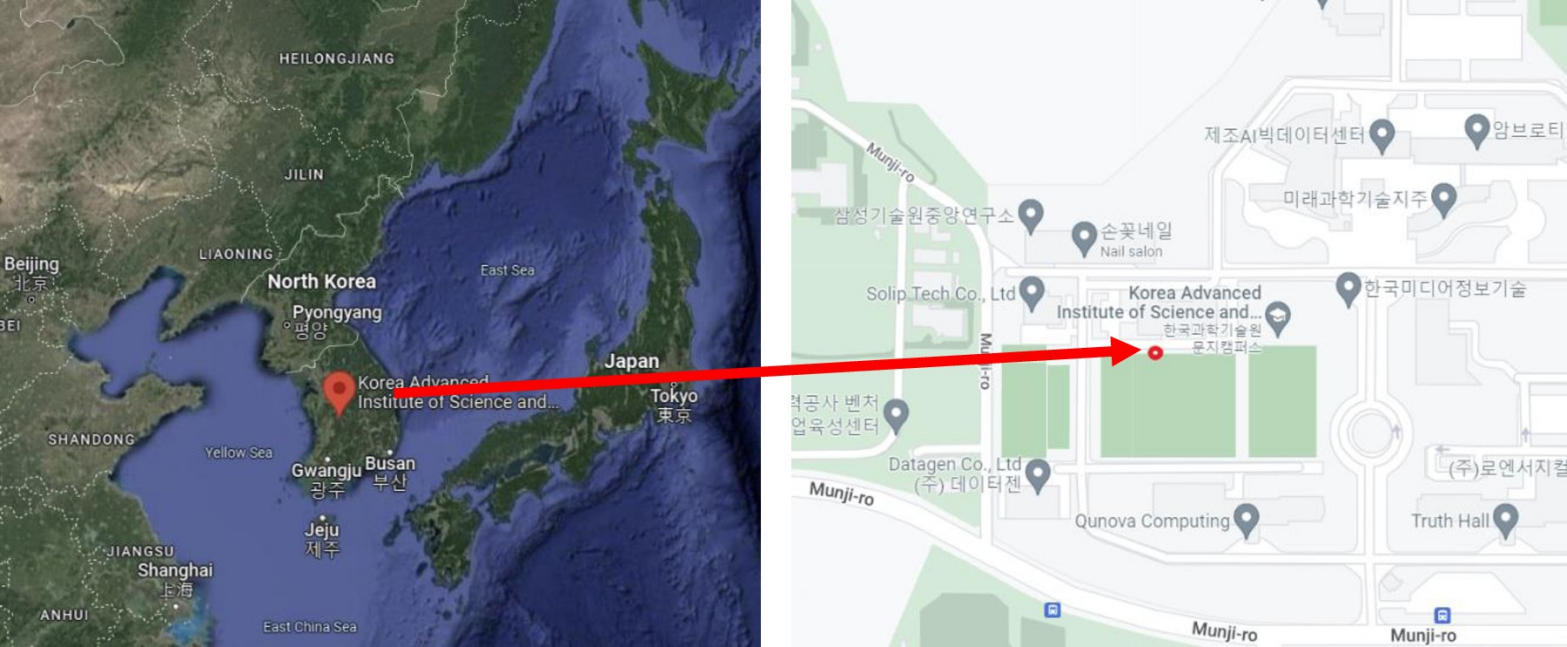
Receiver initial position in WGS-84 coordinate.

To validate the proposed methods, a simulation was performed assuming the receiver moved from normal to harsh conditions to reduce visible satellites. The total simulation time was 250s, input frequency of the satellite navigation measurement data was 2 Hz, and the receiver started at longitude 127.3977°, latitude 36.3919°, and altitude 64.7887m. The initial position was used as the zero position of all the graphs below. The number of early normal visible satellites began with eight (PRN 23, 3, 28, 17, 6, 2, 19, and 9). Fig 2 shows the sky plot of the satellites seen in a normal environment. Fig 3A and Fig 3B, horizontal and vertical positions, respectively, of the receiver moving from the initial position, and Fig 4, the position of the simulation starting point. Based on the data collected in real environment, a simulation was performed assuming a case where the satellite disappears, such as in an urban environment.

Simulations were performed based on the information collected from a real situation. The data were acquired from the u-blox receiver and parrot drone. Table 1 shows the specification of the hardware. The navigation solution was calculated using a single GPS position. The navigation solution was calculated separately without the receiver. The receiver was only used for data collection. The error elements in the simulation reflect real signal errors contained in the collected data. For the simulation, the algorithm was implemented using a numerical analysis program. The raw data that was used includes the satellite ephemeris of each satellite, Doppler values, and carrier measurements from the output of the receiver. The position of the receiver was calculated from the raw data using the least squares method, and the proposed methods were used to continuously calculates the range difference and receiver clock bias in real-time. When a change in the visible satellite is detected owing to the harsh environment, the pseudorange is applied with the algorithm and used to calculate the navigation solution. The application of the algorithm continued until the visible satellites were restored, and when the navigation solution returned to its normal state owing to the recovery of the visible satellite, the algorithm’s application was stopped. To validate the methods and compute the navigation solution, A numerical analysis program, specifically Matlab was used. All of the analysis programs utilized for this study were custom-coded individually, without reliance on any other analytical tools.

**Table 1:** Receiver and drone specification.

Receiver		Drone	
Model	EVK-F9P	Model	Parrot
Channel	L1/L5/E5a	Weight	898 g
Power Supply	5 V	Maximum Flight Time	32 min
Operation Temperature	-40°C to +65°C	Maximum Speed	16 m/s

To verify the error components, ground-based augmentation system (GBAS) data were collected simultaneously during the data collection process. To confirm the simulation situation for a limited time, the navigation solution result was verified by comparing it with the solution using GBAS data. GBAS broadcasts correction information based on data collected from ground stations. When a receiver incorporates this correction data, the receiver’s error is reduced to within 1 meter [26]. Thus, we compared the performance with the receiver’s position when utilizing GBAS correction information.

Table 2 presents the standard deviation of the difference between the GBAS and simulation solutions. The values are in the sub-meter range, and the performance can be verified by applying them.

**Table 2:** Standard deviation of the difference between GBAS solution and simulation solution.

Receiver Position	East-West	North-South	Height	Time
Normal Environment RMS (m)	0.3838	0.4845	0.1312	0.2461

**Fig 5:**
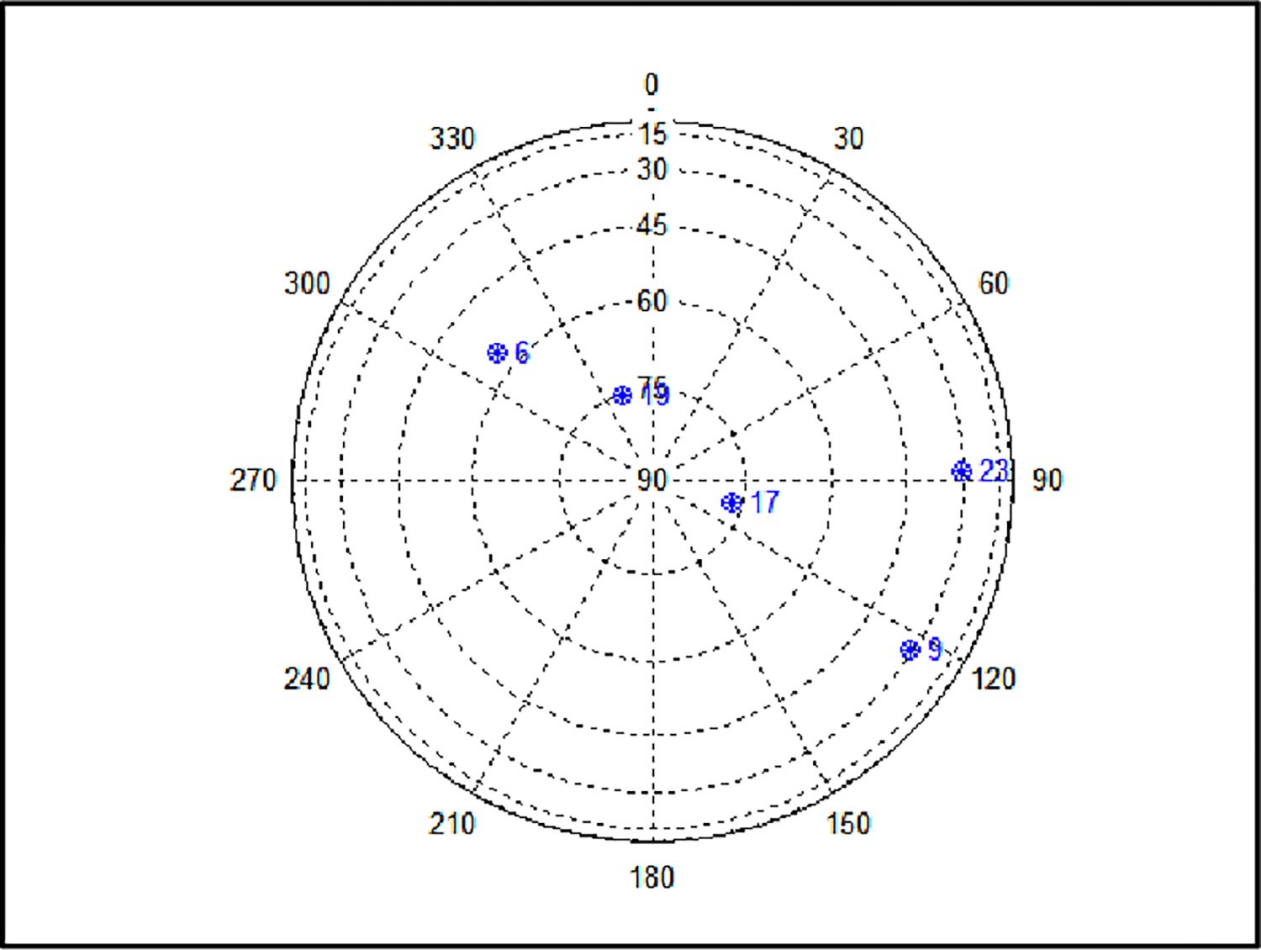
Visible satellite sky plot in harsh environment.

The simulation was set up to enter a harsh environment for 150 s and return to normal within 210 s (for 1 min). Under harsh conditions, the number of visible satellites was reduced to five (PRN 23, 17, 6, 19, and 9) with a mask angle of 30°. Fig 5 shows a sky plot in a scenario wherein the number of visible satellites are reduced owing to harsh environments.

**Fig 6:**
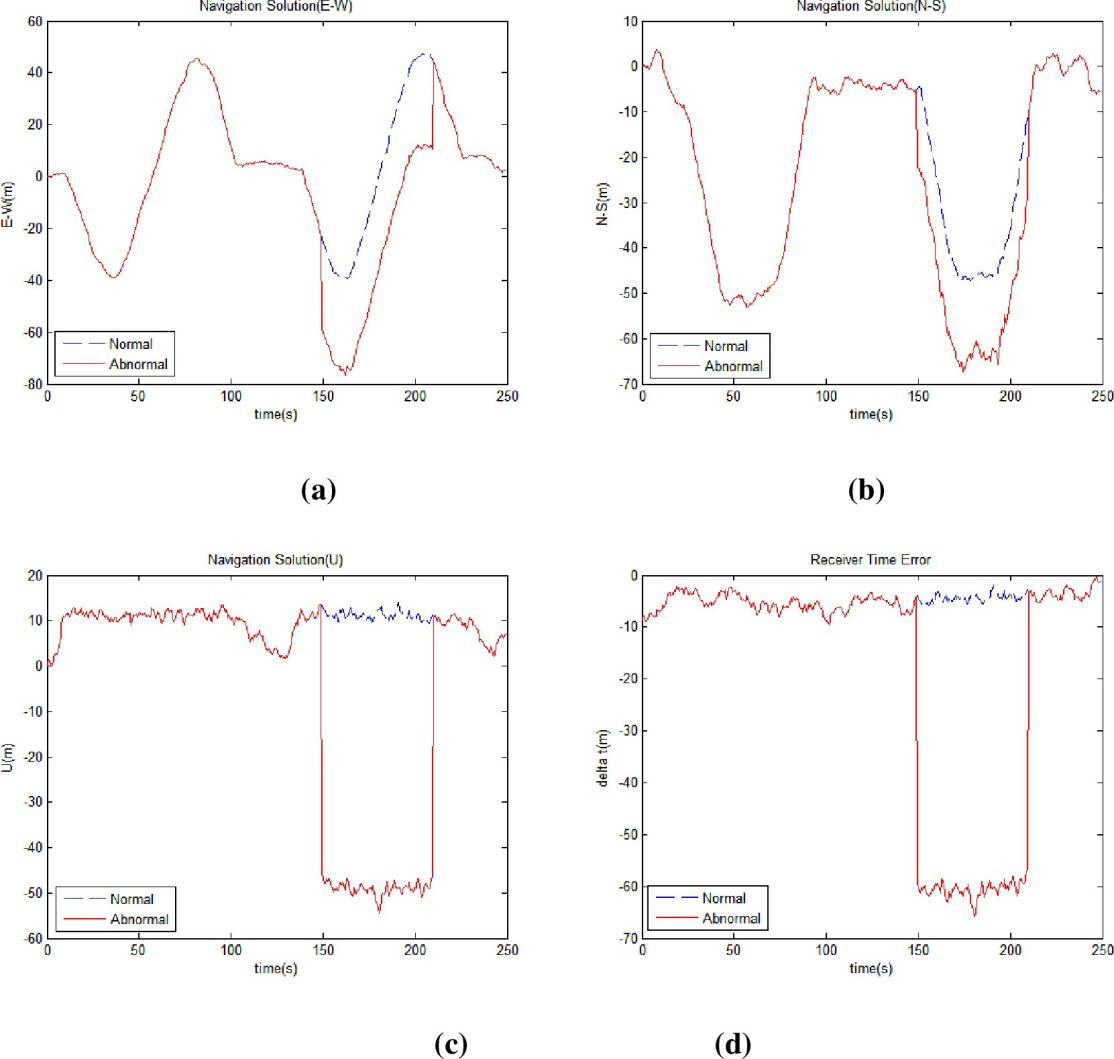
Receiver position and time error in normal and harsh environments. (a) Receiver East-West Position (b) Receiver North-South Position (c) Receiver Altitude (d) Receiver Clock Error.

Fig 6 shows the east-west position (Fig 6A), north-south position (Fig 6B), elevation (Fig 6C), and receiver clock error (Fig 6D) by comparing normal and harsh environment.

Table 3 shows the position errors of the receiver in harsh environment compared to normal environment. Table 4 shows the comparison of DOP in normal and harsh environments. The horizontal and vertical errors were calculated based on the average of the differences in the normal navigation value while entering the harsh environment. Eq (16) is the horizontal error(*E*_*horizon*_) and Eq (17) is the vertical error(*E*_*vertical*_).

**Table 3:** Receiver position errors in harsh environment.

Receiver Position	Horizontal	Vertical
Position Error (m)	39.5882	-60.1913

**Table 4:** DOP in normal and harsh environments.

DOP	GDOP	PDOP	HDOP	VDOP	TDOP
Normal	2.0997	1.8357	1.5794	0.9356	1.0192
Harsh	5.4354	4.5668	3.5673	2.8513	2.9475


$$E_{horizon}=\sqrt{{\left(H_{e\_a}-H_{e\_b}\right)}^{2}+{\left(H_{n\_a}-H_{n\_b}\right)}^{2}}$$ (16)

$$E_{vertical}=V_{u\_a}-V_{u\_b}$$ (17)

where *H*_*e*_*a*_ is east-west error after applying method, *H*_*e*_*b*_ is east-west error before applying method, *H*_*n*_*a*_ is north-south error after applying method, *H*_*n*_*b*_ is north-south before applying method, *V*_*u*_*a*_ is vertical error after applying method, and *V*_*u_b*_ is vertical before applying method. Eq (18) ~ (22) present DOP calculation process.

$$Q={\left[A^{T}A\right]}^{-1}$$ (18)

$$\begin{array}{l} PDOP=\sqrt{q_{11}^{2}+q_{22}^{2}+q_{33}^{2}}\\ TDOP=\sqrt{q_{44}^{2}}\\ GDOP=\sqrt{PDOP^{2}+TDOP^{2}} \end{array}$$ (19)

$$Q_{x}=RQR^{T}$$ (20)

$$\begin{array}{l} HDOP=\sqrt{q_{x11}^{2}+q_{x22}^{2}}\\ VDOP=\sqrt{q_{x33}^{2}} \end{array}$$ (21)

where subscripts indicate elements of a matrix and $R^{T}=[\begin{array}{cccc} n & e & u & 1 \end{array}]$ is matrix including ENU errors.

Simulations show that position errors occur significantly in harsh environments to reduce the number of visible satellites owing to visible satellites reduction. In a harsh environment, the receiver calculates the position using only the measurements of high-elevation satellites, and signals of low-elevation satellites are excluded. The remaining measurements directly influenced the receiver error, which can be seen as a change in the DOP values. The DOP values in Table 4 show a sharp increase due to a mask angle of 30°. When the receiver enters an urban area, rapid changes in position can occur when the building blocks the low-elevation satellites, which affects the navigation control performance. Consequently, the navigation information of the GNSS output cannot be used.

**Fig 7:**
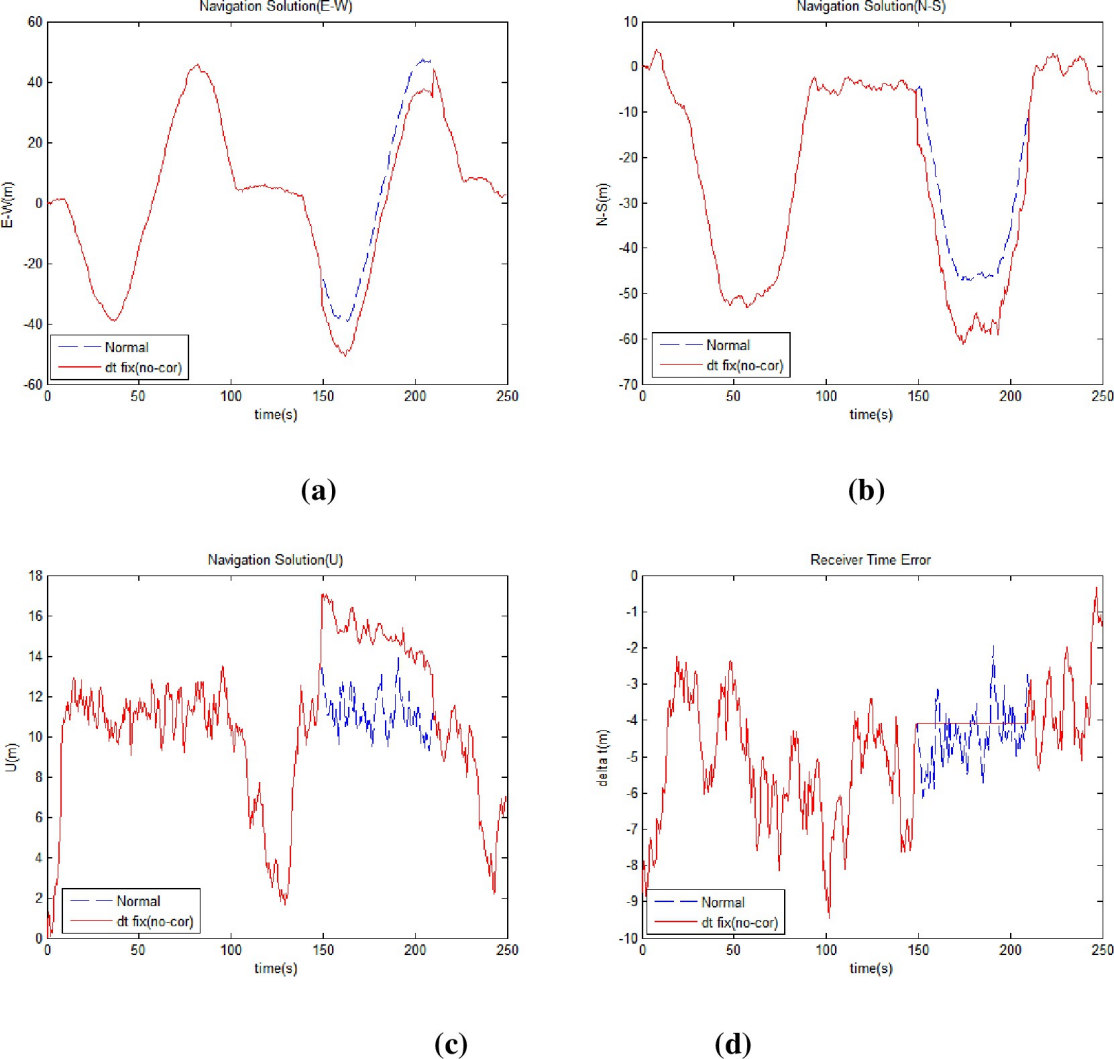
Receiver position and time error applying receiver clock error hold. (a) Receiver East-West Position (b) Receiver North-South Position (c) Receiver Altitude (d) Receiver Clock Error.

The proposed methods were sequentially applied to solve this problem. First, we applied the method of holding the receiver clock error. Fig 7 shows the east-west position (Fig 7A) north-south position (Fig 7B), altitude (Fig 7C), and the receiver clock error (Fig 7D) compared to the normal environment when applying the receiver clock error hold method. Table 5 lists the horizontal position error, vertical position error values, and the improvement rates. Table 6 shows the DOP values, which shows no significant change even when the method is applied considering visible satellites are constant in harsh environments.

**Table 5:** Receiver position error and improvement rates applying receiver clock error hold.

Receiver Position	Horizontal	Vertical
Harsh Environment Position Error (m)	39.5882	-60.1913
Position Error (m)	15.3872	3.8850
Improvement (%)	61.1319	93.5456

**Table 6:** DOP values applying receiver clock error hold.

DOP	GDOP	PDOP	HDOP	VDOP	TDOP
Clock Hold	5.4354	4.5668	3.5673	2.8513	2.9475

While the receiver clock error hold method led to a significant enhancement in vertical error reduction, the overall positional error remained above 15 meters.

**Fig 8:**
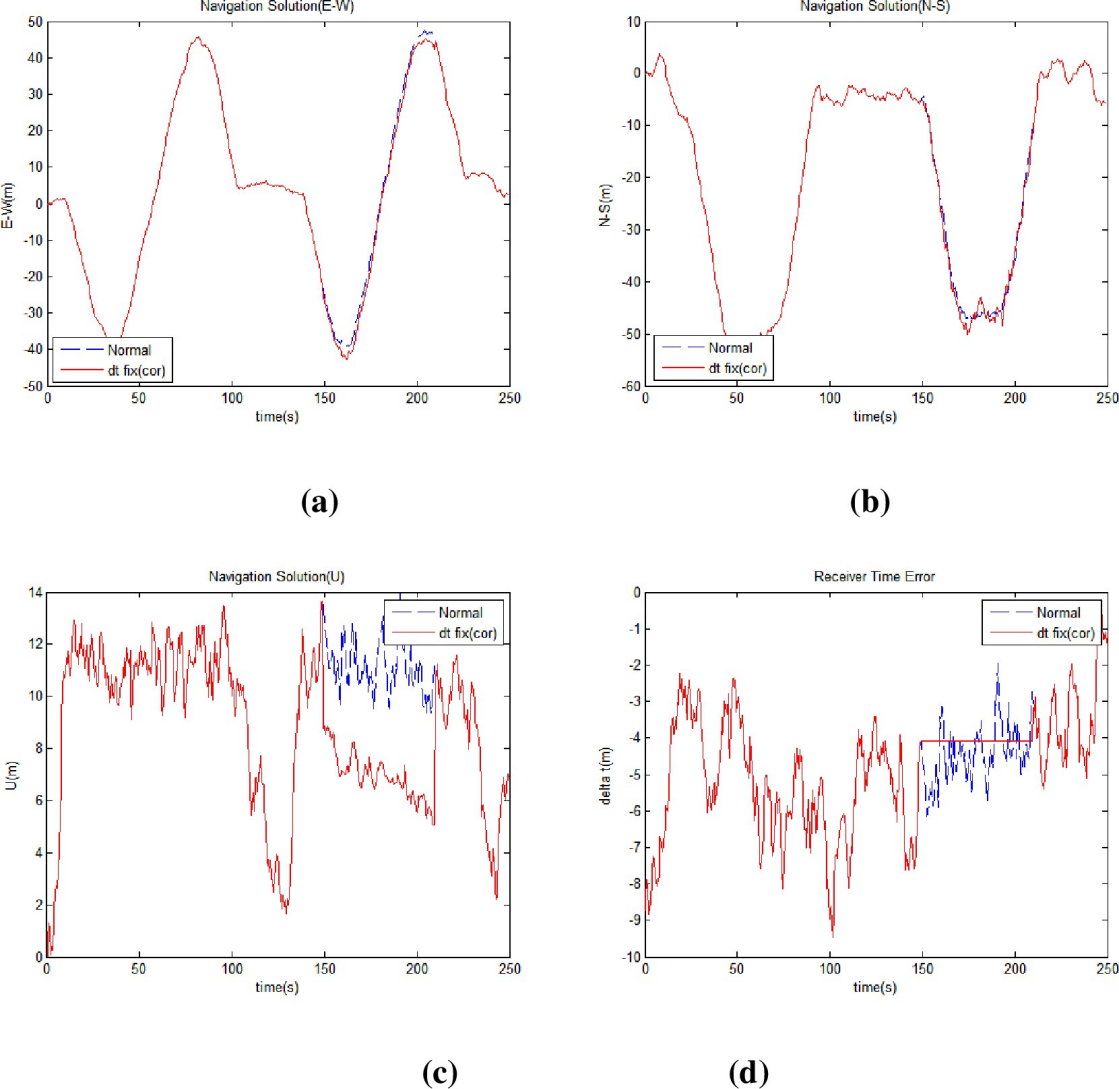
Receiver position and time error applying range difference method. (a) Receiver East-West Position (b) Receiver North-South Position (c) Receiver Altitude (d) Receiver Clock Error.

Then, the proposed range difference method was applied. Fig 8 shows the east-west position (Fig 8A), north-south position (Fig 8B), elevation (Fig 8C), and receiver clock error (Fig 8D) when the receiver clock error hold and range difference methods were applied. Table 7 lists the horizontal position error, vertical position error values and improvement rate relative to the normal environment. Table 8 shows the DOP values when the receiver clock error hold and range difference methods were applied.

**Table 7:** Receiver position error and improvement rates applying range difference method.

Receiver Position	Horizontal	Vertical
Harsh Environment Position Error (m)	39.5882	-60.1913
Position Error (m)	2.6008	-4.2338
Improvement (%)	93.4304	95.9661

**Table 8:** DOP values applying range difference method.

DOP	GDOP	PDOP	HDOP	VDOP	TDOP
Range Difference	5.4354	4.5668	3.5673	2.8513	2.9475

Although the DOP values did not improve because there was no change in the visible satellites, the horizontal position error improved. The improvement rates of the horizontal and vertical position errors were approximately 93%.

**Fig 9:**
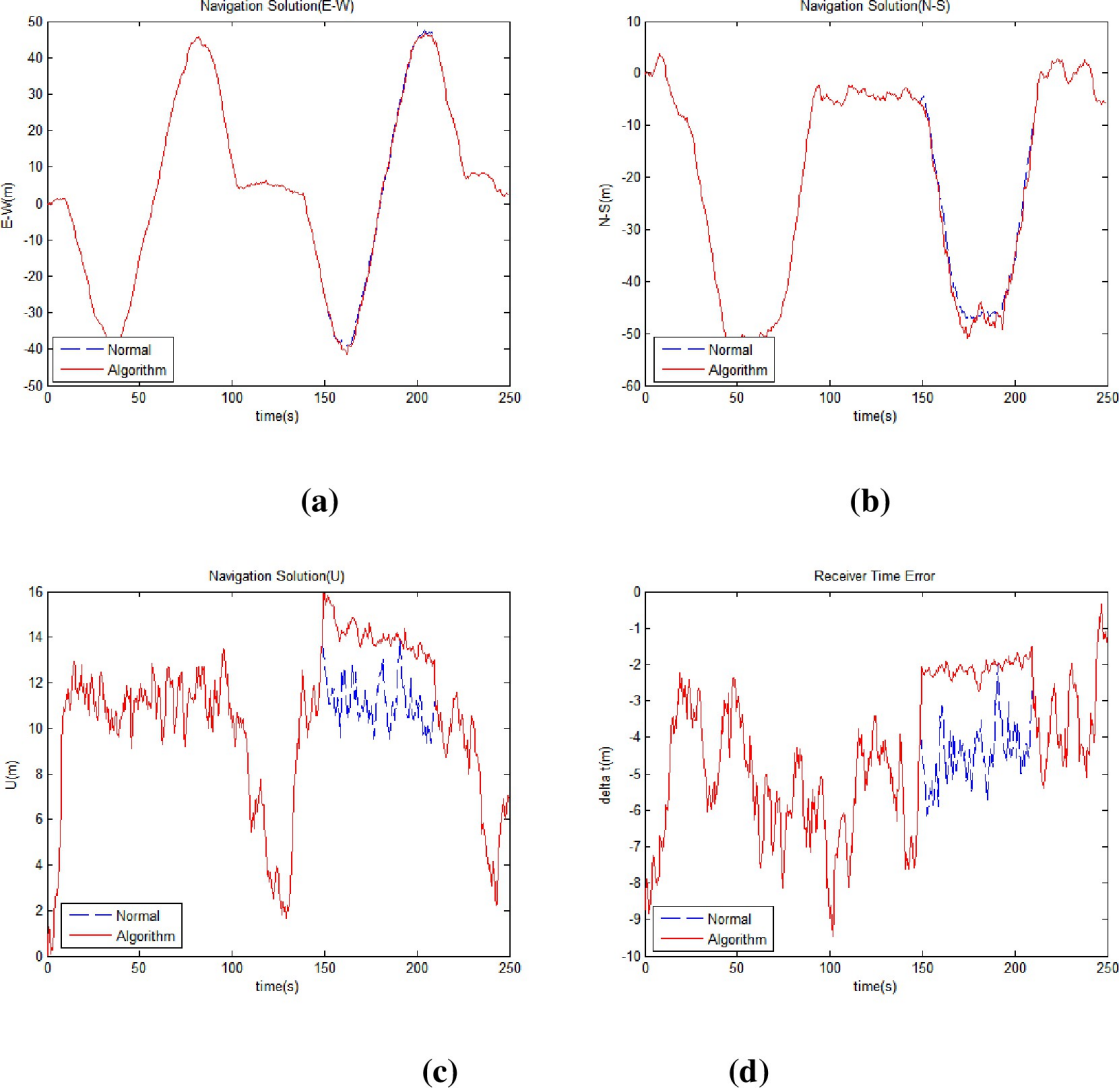
Receiver position and time error applying all proposed methods. (a) Receiver East-West Position (b) Receiver North-South Position (c) Receiver Altitude (d) Receiver Clock Error.

**Fig 10:**
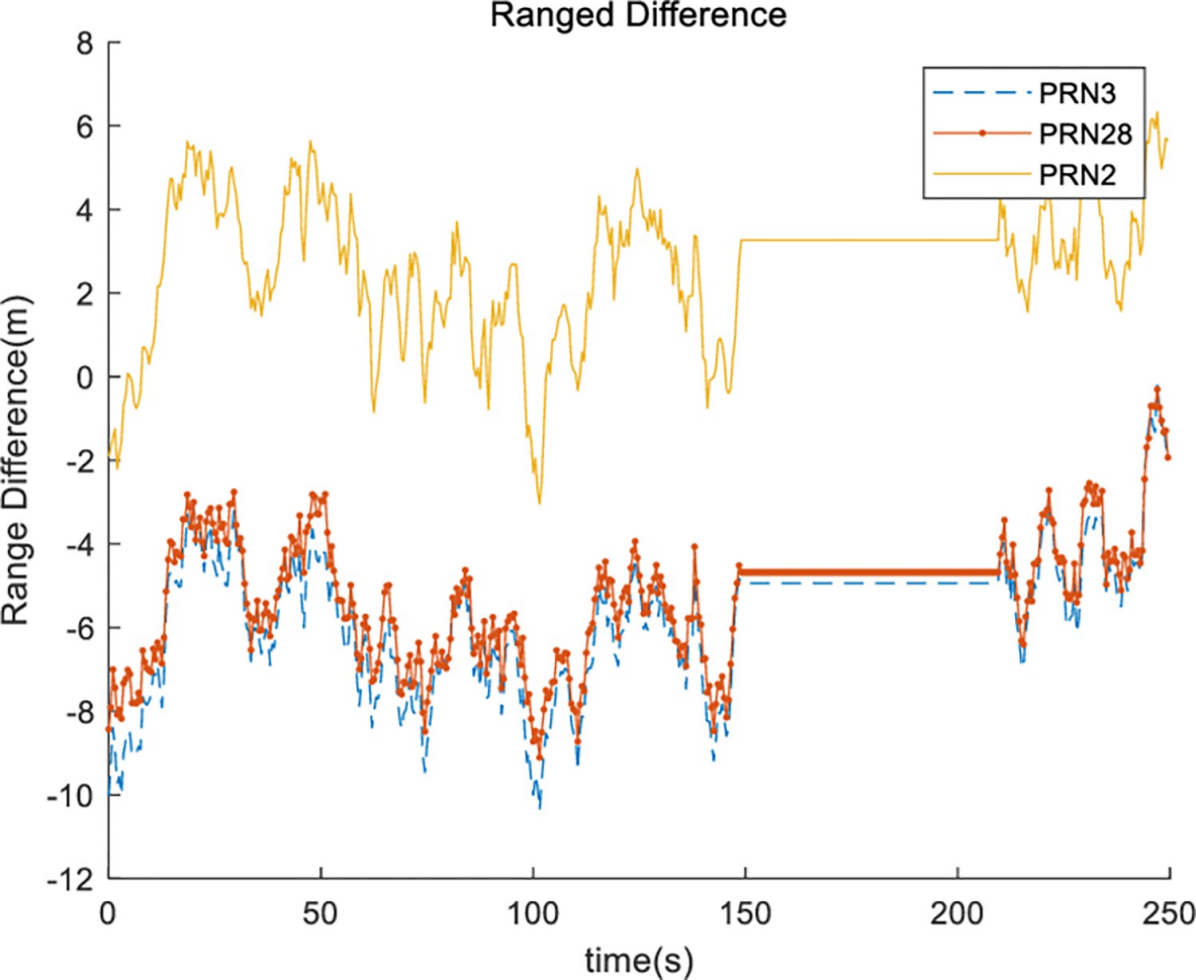
Range difference of satellite with non-line of sight.

Finally, a virtual satellite method was applied, and the navigation solution was recalculated. Fig 9 shows the east-west position (Fig 9A), north-south position (Fig 9B), altitude (Fig 9C), and receiver clock error (Fig 9D) when the proposed virtual satellite method was applied. Table 9 shows the horizontal position error, vertical position error, and improvement rate compared to the normal environment. Table 10 lists DOP values and the improvement rates. The proposed virtual satellite method involves the deployment of a virtual satellite when a satellite signal is blocked in a harsh environment. The position of the virtual satellite is affected by the position of the satellite in its normal state. After applying this method, the satellite arrangement becomes similar to its normal state. Therefore, as shown in Table 10 the DOP values are almost restored to its normal state, and the navigation performance is improved. Fig 10 shows the range difference variation of satellites with NLOS (Non-Line of Sight) when the proposed methods are applied.

**Table 9:** Receiver position error and improvement rates applying all proposed methods.

Receiver Position	Horizontal	Vertical
Harsh Environment Position Error (m)	39.5882	-60.1913
Position Error (m)	2.1146	2.8816
Improvement (%)	94.6585	95.2126

**Table 10:** DOP values and improvement rates applying all proposed methods.

DOP	GDOP	PDOP	HDOP	VDOP	TDOP
Normal	2.0997	1.8357	1.5794	0.9356	1.0192
Harsh	5.4354	4.5668	3.5673	2.8513	2.9475
All Methods	2.1056	1.8403	1.5830	0.9385	1.0231
Improvement (%)	99.8231	99.8316	99.8189	99.8486	99.7978

When all the proposed methods were applied, the accuracy improved by approximately 95%, with horizontal and vertical errors of 2.1146 m and 2.8816 m, respectively. Furthermore, over 99% improvements were made in terms of the DOP value, indicating that the DOP performance improved with the virtual satellite arrangement. The performance improvement of DOP in Table 10 shows how much it recovers from the value after the satellite disappeared to the DOP value before it disappeared. Eq (22) shows the calculation of DOP performance improvement.


$$improvement_{DOP}=\frac{\left|harsh-method\right|}{\left|harsh-normal\right|}\times 100$$ (22)

**Fig 11:**
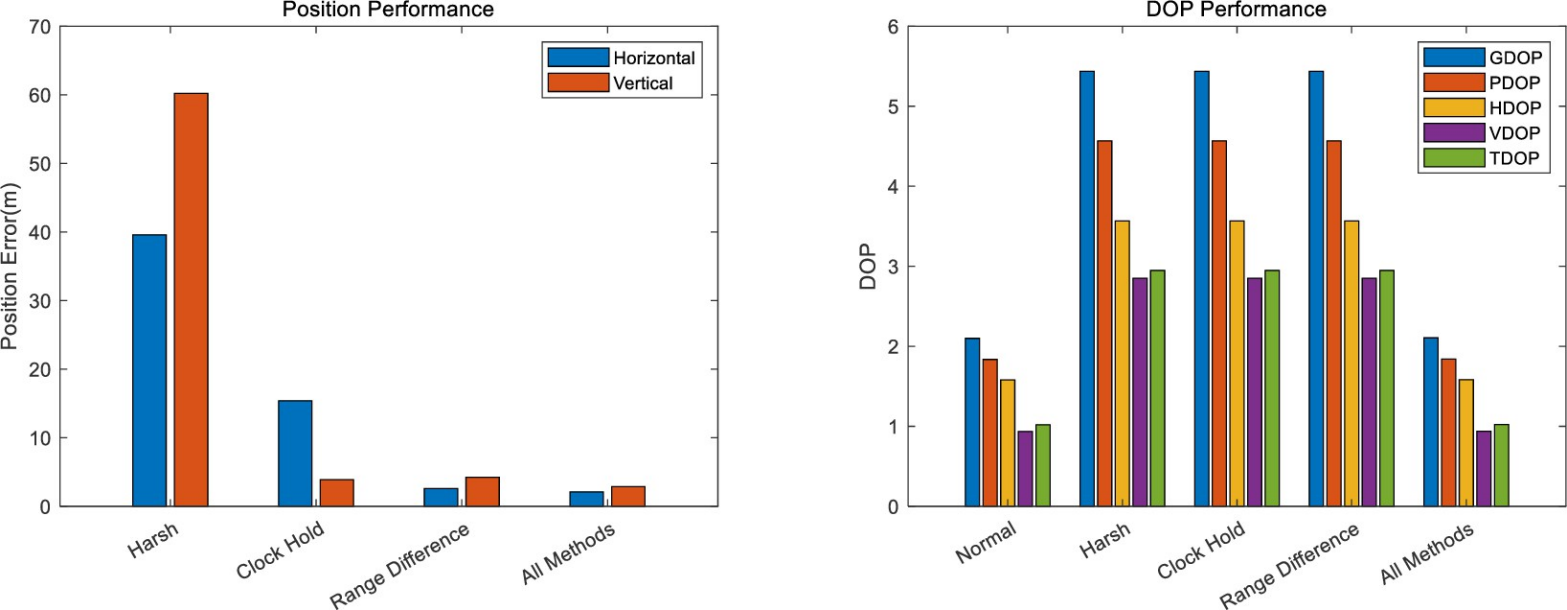
Performance improvement. (a) Position (b) DOP.

Fig 11 is a summary of performance improvements. When the proposed technique is applied, the improvement effect of position performance and DOP performance is shown. As all the proposed methods were applied sequentially, the effect of performance improvement approached the normal level.

## Discussion

In the simulation results, we confirmed that the proposed methods: range difference, receiver clock error hold, and virtual satellite, enhance navigation performance. In urban areas, GNSS signals are frequently blocked by tall buildings and city facilities. Because a GNSS receiver calculates the position and time information by receiving navigation signals from satellites, the positioning performance is degraded with limited signals. Therefore, it is difficult to use GNSS for systems that require high navigation performance, such as unmanned aerial vehicles or autonomous vehicles in urban areas. Therefore, an auxiliary sensor must be used to obtain accurate position information. In this study, the navigation performance was improved by the range difference, clock error hold, and virtual satellites when calculating the navigation solution of the satellite navigation receiver introducing auxiliary sensors, which was confirmed through simulation. When the number of satellites that can receive signals decreases when entering a harsh environment, the navigation performance in both horizontal and vertical positions deteriorates, which verifies the difficulties in suing the satellite navigation system as it is in a system requiring high reliability.

GNSS measures the distance between the satellite and receiver and calculates the position of the receiver by triangulation using the position and distance of the satellite. At this time, the pseudorange measurement is calculated using the difference between the departure time and arrival time of the signal. Because the satellite clock and receiver clock are not synchronized, the receiver clock error is also included in the calculation equation. Therefore, the receiver clock error also changes according to the number of visible satellites; however, it does not change significantly in a short period of time. With this point in mind, this study applied a technique to hold the clock error. The simulation confirmed that the performance was improved compared with the normal state.

Additionally, the range difference is related to the distance between the receiver and satellite. GNSS uses the least-squares method from four or more satellite signals. At this time, the distance between the receiver and satellite is different from the measured pseudorange, and this difference also changes when the number of satellites changes. The range difference before entering a harsh environment is more suitable for the real environment than the range difference in a harsh environment. The navigation performance can be improved if this range difference is used, even in harsh environments. The simulation confirmed that the performance was further improved when the range difference method was applied.

Finally, virtual satellites were applied. Deterioration in navigation performance is a phenomenon caused by a decrease in the number of visible satellites. The navigation solutions which are calculated only with satellites near the zenith, deteriorate the DOP performance. Even if a small range error occurs, it acts as a large error component in the navigation solution. In this study, the DOP and navigation solution performances were improved by arranging virtual satellites in a given navigation solution and calculating the navigation solution with feedback. Finally, the performance improvement was confirmed by applying all the proposed techniques.

These methods prevent sudden position changes in harsh environments such as urban areas where satellite signals are blocked. The continuity of the navigation solution was maintained even when the number of visible satellites decreased. The navigation performance recovered in a normal environment. Thus, the proposed methods have the performance of reducing navigation error in harsh environment without assistant sensors.

The techniques proposed in this study focus on preventing the deterioration of navigation performance by using the situation before the signal from the navigation satellite is blocked to calculate the navigation solution of the receiver. In this study, performance degradation was prevented even if the satellite navigation receiver entered a harsh environment with only satellite navigation receiver measurements, without additional sensors or devices. If these techniques are applied, the reliability of GNSS can be improved in urban areas and a stable location service can be provided. Simulations to validate the proposed method were conducted under a limited time constraint. In future works, simulations reflecting long-term changes in satellite conditions and various urban environments can be conducted for the overall performance analysis.

The proposed method represents a technique aimed at averting the degradation of navigation performance, even in instances of deteriorating navigation signal reception environments. Unlike previous methods that primarily sought to enhance signal availability by increasing the number of satellites, the proposed method adopts a different strategy. It continuously monitors historical navigation signals and derives receiver measurements by estimating the conditions as they existed before environmental changes occurred. By preserving the receiver’s previous performance, the proposed method significantly contributes to the enhancement of navigation performance, even in challenging reception conditions. This maintenance of previous performance has the added benefit of bolstering system stability by preventing abrupt positional variation, particularly in harsh environments like urban areas.

Nevertheless, the proposed methods rely on data obtained before entering harsh environments, introducing the potential issue of performance deterioration if NLOS conditions persist. Over an extended duration, there is a likelihood that measurement errors may accumulate, even among visible satellites, potentially leading to degradation in navigation performance. In future research, we may explore methods to address performance degradation by comparing the results of the proposed method with those obtained using only the currently visible satellites.

## Conclusions

GNSS signals are blocked by obstacles in urban areas or high mountain canyons, causing severe position errors in the receiver. To obtain navigation solutions using least-square methods with available measurements, performance degradation is inevitable. This study proposed range difference, receiver clock error hold, and virtual satellite methods to maintain the navigation solution performance despite the lack of visible satellites.

The proposed method was developed to maintain the navigation performance in harsh environments by maximally maintaining normal conditions before the receiver enters harsh environments. Even in harsh environments, where the satellite signal is blocked, the measurement of each satellite receiving the signal is not different from the measurement of the normal state. Errors occur owing to the characteristics of satellite navigation, which uses several satellite signals simultaneously. Considering this point, when entering a harsh environment, the navigation performance can be maintained using previous information such as the range difference of each measured value and the receiver clock error in the normal state. Simulations confirmed that the navigation performance was maintained, proving it to a useful method in harsh environments. Additionally, it can prevent errors in the control input by preventing sudden changes in the output of the receiver. The general use of the proposed methods can improve the navigation performance through software replacement. There may be various methods for obtaining navigation information in an urban area. However, GNSS can calculate a navigation solution only by receiving satellite signals even without special initial information or additional information. If the GNSS navigation performance is maintained in downtown areas where signals are blocked, diversity in obtaining navigation information can be secured, which can contribute toward expanding the applications of GNSS by improving the robustness of satellite navigation systems. In the future, research can be conducted on various environmental changes. Methods can be applied to the receiver, experiments can be performed in a real long-term environment, and the practical applicability of the methods will be confirmed.

## Supporting information

S1 Data (XLSX)

S2 Data (XLS)

S3 Data (XLS)

S4 Data (XLS)

S5 Data (XLS)

S6 Data (XLS)

## References

[1] Isik OK, Hong J, Petrunin I, Tsourdos A. Integrity Analysis for GPS-Based Navigation of UAVs in Urban Environment. Robotics. 2020;9(3): 66.

[2] Xie P, Petovello MG. Measuring GNSS Multipath Distributions in Urban Canyon Environments. IEEE Transactions on Instrumentation and Measurement. 2015;64(2): 366–377.

[3] Park SG, Cho DJ. A Performance Improvement on Navigation Applying Measurement Estimation in Urban Weak Signal Environment. Journal of the Korea Institute of Information and Communication Engineering. 2014;18(11): 2745–2752.

[4] Tay S, Marais J. Weighting models for GPS Pseudorange observations for land transportation in urban canyons. 6th European Workshop on GNSS Signals and Signal Processing. 2013. Germany. hal-00942180: 1–4.

[5] Groves P. Shadow Matching: A New GNSS Positioning Technique for Urban Canyons. Journal of Navigation. 2011; 64(3): 417–430. doi: 10.1017/S0373463311000087

[6] Wang L, Groves P, Ziebart M. GNSS Shadow Matching: Improving Urban Positioning Accuracy Using a 3D City Model with Optimized Visibility Scoring Scheme. Navigation. 2013;60(3): 195–207. doi: 10.1002/navi.38

[7] Wang L, Groves P, Ziebart M. Smartphone Shadow Matching for Better Cross-street GNSS Positioning in Urban Environments. Journal of Navigation. 2015;68(3): 411–433. doi: 10.1017/S0373463314000836

[8] Yozevitch R, Moshe B, Weissman A. A Robust GNSS LOS/NLOS Signal Classifier. Navigation. 2016;63(4): 429–442. doi: 10.1002/navi.1662016

[9] Borio D, Closas P. Robust transform domain signal processing for GNSS. Navigation. 2019;66(2): 305–323. doi: 10.1002/navi.300

[10] Adjrad M, Groves PD. Intelligent Urban Positioning using Shadow Matching and GNSS Ranging Aided by 3D Mapping. Proceedings of the 29th International Technical Meeting of the Satellite Division of The Institute of Navigation (ION GNSS+ 2016). 2016. USA. 534–553.

[11] Crespillo OG, Andreetti A, Grosch A. Design and Evaluation of Robust M-estimators for GNSS Positioning in Urban Environments. 2020 International Technical Meeting of The Institute of Navigation. 2020. USA. doi: 10.33012/2020.17211

[12] Yozevitch R, Ben-Moshe B, Dvir A. GNSS Accuracy Improvement Using Rapid Shadow Transitions. IEEE Transactions on Intelligent Transportation Systems. 2014;15(3): 1113–1122.

[13] Groves PD. Jiang Z. Wang L. Ziebart MK. Intelligent Urban Positioning using Multi-Constellation GNSS with 3D Mapping and NLOS Signal Detection. Proceedings of the 25th International Technical Meeting of the Satellite Division of The Institute of Navigation (ION GNSS 2012). 2012. USA. 458–472.

[14] Momoh JA, Bhattarai S, Ziebart M. Receiver clock jump and cycle slip correction algorithm for single-frequency GNSS receivers. GPS Solutions. 2019;23(38). doi: 10.1007/s10291-019-0832-4

[15] Kim I, Park C, Jee G, Lee JG. GPS Positioning Using Virtual Pseudorange. Control Engineering Practice. 1998;6(1): 25–35.

[16] Won D, Ahn J, Lee S, Lee J, Sung S, Park H, et al. Weighted DOP with Consideration on Elevation-Dependent Range Errors of GNSS Satellites. IEEE TIM (Transaction on Instrumentation and Measurement). 2012;61(12): 3241–3250.

[17] Li X, Ge M, Dai X. et al. Accuracy and reliability of multi-GNSS real-time precise positioning: GPS, GLONASS, BeiDou, and Galileo. Journal of Geodesy. 2015;89: 607–635. doi: 10.1007/s00190-015-0802-8

[18] Li X, Zhang X, Ren X, et al. Precise positioning with current multi-constellation Global Navigation Satellite Systems: GPS, GLONASS, Galileo and BeiDou. Scientific Reports. 2015;5: 8328 doi: 10.1038/srep08328 25659949 PMC4321187

[19] Montenbruck O, Steigenberger P. Hauschild A. Broadcast versus precise ephemerides: a multi-GNSS perspective. GPS Solutions. 2015; 9: 321–333.

[20] Jin S, Wang O, Dardanelli G. A Review on Multi-GNSS for Earth Observation and Emerging Applications. Remote Sensing. 2022;14(16): 3930. doi: 10.3390/rs14163930

[21] Baybura T, Tiryakioğlu I, Uğur M, Solak H, Şafak S. Examining the Accuracy of Network RTK and Long Base RTK Methods with Repetitive Measurements. Journal of Sensors. 2019; 3572605 doi: 10.1155/2019/3572605

[22] Dardanelli G, Maltese A, Pipitone C. Pisciotta A, Brutto M. NRTK, PPP or Static, That Is the Question. Testing Different Positioning Solutions for GNSS Survey. Remote Sensing. 2021;13(7): 1406. doi: 10.3390/rs13071406

[23] Zhang B, OdijkA D. Method for processing GNSS data from regional reference networks to enable single-frequency PPP-RTK. Chinese Journal of Geophysics. 2015;58(7): 2306–2319. doi: 10.6038/cjg20150709

[24] Song C, Ahn J, Choi M, Jang J, Heo M, and Lee Y. Virtual Satellite and Virtual Range Measurement Generation for the GNSS Position Accuracy Improvement. Journal of the Korean Society for Aeronautical & Space Sciences. 2017;45(9): 757–765.

[25] Kovalevskyi E, Using virtual satellites for navigation definitions. AEROSPACE SYSTEMS FOR MONITORING AND CONTROL. 2008;36(3): 95–98.

[26] Enge P, Local area augmentation of GPS for the precision approach of aircraft. Proceedings of the IEEE, 1999;87(1): 111–132.

